# The Need for a Revision of Fluoroquinolone Breakpoints for Interpretation of Antimicrobial Susceptibility Testing of Feline Bacterial Isolates

**DOI:** 10.1111/jvp.70028

**Published:** 2025-09-29

**Authors:** Mark G. Papich, Lacie A. Gunnett, Marilyn N. Martinez

**Affiliations:** ^1^ College of Veterinary Medicine North Carolina State University Raleigh North Carolina USA; ^2^ Zoetis Kalamazoo Michigan USA; ^3^ Center for Veterinary Medicine, US Food and Drug Administration Rockville Maryland USA

**Keywords:** antimicrobial, cats, enrofloxacin, marbofloxacin, susceptibility testing

## Abstract

The fluoroquinolone antimicrobial agents, enrofloxacin and marbofloxacin, were approved in the United States for cats in 1990 and 2001, respectively. In 2023, revised breakpoints for testing isolates from dogs were published. These canine breakpoints are discordant with the current feline breakpoints. This study was aimed at suggesting new feline breakpoints using a pharmacokinetic‐pharmacodynamic (PK‐PD) approach and new pharmacokinetic data. The PK‐PD derived cutoff values (CO_PD_) were compared to microbiologic data available for testing the susceptibility of targeted pathogens since the original approval. Compared to the current Clinical and Laboratory Standards Institute (CLSI) breakpoints for enrofloxacin and marbofloxacin in cats, these revised breakpoints are lower by two dilutions for the Enterobacterales, *Pseudomonas aeruginosa, Staphylococcus* spp., and *Pasteurella multocida* . Isolates that may have previously tested susceptible (S) may test resistant (R) using these suggested breakpoints. We also are suggesting a susceptible dose‐dependent (SDD) category for testing marbofloxacin against these isolates from cats that allows for a higher dose. These suggested breakpoints may be considered by laboratories, standard‐setting organizations, and industry sponsors of these antimicrobials for testing common bacteria isolated from cats.

## 1 Introduction

The fluoroquinolones enrofloxacin (Baytril) and marbofloxacin (Zeniquin) were approved for use in cats by the U.S. Food and Drug Administration (FDA) in December 1990 and June 2001, respectively. In 2023, the breakpoints for testing isolates from dogs for these antimicrobials were revised (Papich et al. 2023). These breakpoints were substantially lower than the previously published breakpoints in earlier editions of the Clinical and Laboratory Standards Institute (CLSI) Veterinary Antimicrobial Susceptibility Testing (VAST) VET01(S) document. Because the revised canine breakpoints are discordant with the current feline breakpoints, a revision of the feline breakpoints using pharmacokinetic‐pharmacodynamic (PK‐PD) relationships for fluoroquinolones is needed. It is particularly important to develop new enrofloxacin breakpoints for a dose that is limited to 5 mg/kg per day in cats. Higher doses have been associated with retinal injury and blindness in cats (Baytril product label, Elanco, U.S.). This analysis demonstrates the need for revised breakpoints and includes pharmacokinetic and microbiologic data that were not available when the original breakpoints were approved.

The original approved label for enrofloxacin in cats was indicated for the treatment of dermal infections (wounds and abscesses) caused by susceptible strains of *Escherichia coli* , *Klebsiella pneumoniae* , *Proteus mirabilis* , and *Staphylococcus aureus*, respiratory infections (pneumonia, tonsillitis, rhinitis) caused by susceptible strains of *Escherichia coli* and *Staphylococcus aureus* , and urinary cystitis caused by susceptible strains of *Escherichia coli, Proteus mirabilis*, and *Staphylococcus aureus* . The dose on the label was 2.5 mg/kg twice daily and in June 1997 expanded to 5–20 mg/kg per day. However, in February 2001, the label was revised to lower the dose in cats to 5 mg/kg either as a single daily dose or divided into two equal 2.5 mg/kg doses administered every twelve (12) hours because of post‐marketing adverse drug experience information that identified retinal injury in cats receiving doses exceeding 5 mg/kg per day.

The approved label for oral marbofloxacin in cats is a dose range of 2.8–5.5 mg/kg with an indication for the treatment of skin and soft tissue infections. This approval provided no label information on the bacteria that would be susceptible to marbofloxacin within this dose range.

The CLSI subcommittee approved methods and breakpoints for testing susceptibility of bacterial isolates from cats to enrofloxacin and marbofloxacin in 1999 and 2004, respectively. Those interpretive categories and breakpoints appear in the 7th edition of the CLSI standard document VET01S (CLSI 2024), but new or revised breakpoints have not been published.

If accurate breakpoints are not used for susceptibility testing, there is a high likelihood of failure to meet the optimum PK‐PD target when these agents are administered to cats in accordance with the most current approved labels. If the PK‐PD target is not achieved, the resulting suboptimal exposure may lead to the selection of resistant strains that multiply and become the dominant population in an infection (Martinez et al. 2012; Drusano 2004).

Our objective for this study was to present an updated and revised analysis of the current enrofloxacin and marbofloxacin breakpoints for bacteria isolated from cats. These suggested breakpoints may be considered by laboratories, standard‐setting organizations, and industry sponsors of these antimicrobials for testing common bacteria isolated from cats. Updated breakpoints may improve the safe and effective use of fluoroquinolones in the treatment of feline bacterial infections.

## 2 Methods

We primarily used pharmacokinetic data and pharmacokinetic‐pharmacodynamic (PK‐PD) analysis to examine the breakpoints for enrofloxacin and marbofloxacin to test common bacteria isolates from cats. Other standard‐setting organizations (USCAST, The National Antimicrobial Susceptibility Testing Committee for the United States 2018) and CLSI (CLSI 2021) have used the PK‐PD cutoff (CO_PD_) as the primary determinant of the clinical breakpoint. We also compared the CO_PD_ to microbiology data (MIC distributions) to explore the relationship of the suggested breakpoint to the MIC distribution for common bacteria isolated from cats.

### 2.1 Pharmacokinetic Data

Pharmacokinetic data for enrofloxacin were obtained from published references (Seguin et al. 2004; Richez et al. 1997), data generated by the sponsor published on the approved label, and Freedom of Information (FOI) Summaries, available from the FDA for six generic products that are bioequivalent to the innovator product. The FOI Summaries containing pharmacokinetic data for these approved products are available from the Animal Drugs at FDA website (https://animaldrugsatfda.fda.gov/adafda/views/#/search). The detailed pharmacokinetic results are presented in Tables S1–S7, which provide a summary of the relevant pharmacokinetic data for each study, the number of cats in each study, and the dose administered. For published studies, the mean and standard deviation (Std Dev) are presented from the published study. For the generic drugs, the mean area‐under‐the‐curve (AUC) and peak concentration (C_MAX_) were reported in the FOI Summary data with the upper and lower bounds for the 90% confidence intervals (CI). We converted the CI values to a coefficient of variation (CV%) and Std Dev to obtain a measure of variability for our tables. The method used for this conversion is provided in Tables S1–S7. This analysis produced an estimate of within‐subject variability. In the absence of additional sources of between‐subject variability information, we combined these values, along with the inter‐study determination of Least Square Means (LS mean) and Std Dev (which included data on between‐subject variability) to provide the Std Dev value required as input for the Monte Carlo simulations. We acknowledge that an underestimation of the between‐subject variability associated with our method for estimating variability will lead to a higher rather than lower estimate of the PK‐PD cutoff value (CO_PD_) (Toutain et al. 2023). However, we considered it necessary to include as much data as is available, even though the variability estimate may be somewhat lower than observed across the patient population.

To determine the total mean and variability associated with the systemic clearance value (CL/F) across all investigations, values were weighted for differences in the number of subjects per study. To obtain an unbiased estimate of averages and variability in the face of study imbalance, the LS mean, and its associated variability, was calculated (Martinez and Bartholomew 2017). To calculate the variability in the LS means, the sums of squares were determined within each study, multiplying the squared Std Dev of that investigation by its corresponding degrees of freedom. This reflected the within‐study sums of squares. Between‐study sums of squares likewise reflected the squared difference between the cross‐study LS mean value and the mean for the investigation. Within each study, this estimate was multiplied by the number of observations. For the total variability, the within‐ and between‐study variability were added, and the sum was then divided by the overall degrees of freedom (defined as total number of observations (*N*) across all studies) (Martinez and Bartholomew 2017).

For the generic drug studies, CL/F was not provided in the data tables and therefore needed to be calculated from the available pharmacokinetic data and the administered dose according to the following formula: CL/F = Dose/AUC, where CL/F is the clearance per fraction absorbed from the oral dose (L/kg/h), and AUC is the total area‐under‐the‐curve from time zero to infinity.

Enrofloxacin is metabolized to the active metabolite ciprofloxacin, which contributes to microbiological activity with additive effects (Lautzenhiser et al. 2001; Blondeau et al. 2012). Therefore, both enrofloxacin and ciprofloxacin were factored into our assessments of total exposure of active drug moieties. The extent of conversion from enrofloxacin to ciprofloxacin (percent ciprofloxacin contribution to the total fluoroquinolone AUC) was obtained from three published studies (Richez et al. 1997; Foster et al. 2023; Kordick et al. 1997) and averaged across the studies to produce the mean and variability of the percent contribution used in the simulations.

Marbofloxacin pharmacokinetic data from cats were obtained from one published study (Albarellos et al. 2005), the manufacturer's original data available for the approved product in the FOI Summary (NADA 141–151), and FOI Summary data from three approved bioequivalent generic products obtained from the FDA website listed above for Animal Drugs at FDA. We calculated the necessary pharmacokinetic parameters and their variability from the marbofloxacin data in the FOI Summary as described above for enrofloxacin.

### 2.2 Protein Binding

As only the unbound antimicrobial fraction (*fu*) is considered biologically active, breakpoint development must consider the amount of protein binding for the antimicrobial agent. To properly perform PK‐PD analysis for the fluoroquinolone antimicrobial agents, only the unbound drug was used for model simulations (protein unbound, or fraction unbound) for each drug analyzed. We used protein binding data from a published pharmacokinetic study in cats for enrofloxacin (Bregante et al. 2003). For marbofloxacin, the only protein binding data available for cats was from a sponsor's unpublished technical report. However, this report listed only a single value with no description of the method, concentrations analyzed, or variability. Therefore, one of our authors (MGP) performed an independent marbofloxacin protein binding study with feline plasma in the laboratory using the same method we have used previously for analysis of marbofloxacin in canine plasma (Bidgood and Papich 2005). Our method used a filtration device to separate the protein‐bound drug fraction from the unbound fraction (Centrifree Micropartition System, Amicon, Beverly, MA). A fortified 500 μL plasma sample was loaded into the top chamber of the filtration device, incubated at 37°C for 30 min, and centrifuged using a fixed‐angle rotor at 1500 ***g*** at 37°C. The protein‐free ultrafiltrate was collected in the bottom reservoir of the filtration device and analyzed using high‐pressure liquid chromatography (HPLC) with fluorescence detection with a method used previously and validated in our laboratory (Bidgood and Papich 2005). For this analysis in feline plasma, we tested pooled feline plasma fortified (spiked) with marbofloxacin at three concentrations to represent the low (1.25 μg/mL), medium (2.5 μg/mL), and high (5.0 μg/mL) of reported concentrations. We tested three replicates at each concentration. Percent protein binding was derived from the following formula (Equation 1):

(1) $$\%\text{Protein Binding}=\frac{\left[\text{Drug Concentration}\right]-\left[\text{Concentration in Filtrate}\right]}{\left[\text{Drug Concentration}\right]}\times 100$$

where the Drug Concentration is the fortified concentration before centrifugation, and Concentration in Filtrate is the concentration in the filtrate collected after centrifugation.

The mean and variability (Std Dev) of protein binding measurements for each drug were entered into the Monte Carlo Simulations (described below).

### 2.3 Monte Carlo Simulations and Probability of Target Attainment (PTA)

Monte Carlo simulations were used to determine the probability of target attainment (PTA) to reach PK‐PD targets for a range of bacteria MIC values. Monte Carlo simulations are used by clinical investigators to determine if antimicrobial dose regimens can reach therapeutic targets with a high probability (Ambrose 2006). The application of this approach to derive antimicrobial susceptibility testing breakpoints was reviewed by others (Ambrose 2006; Turnidge and Paterson 2007).

Probability of PK‐PD target attainment (PTA) for marbofloxacin and the combination of enrofloxacin plus ciprofloxacin was calculated for a 24‐h dose interval by use of the free drug area‐under‐the‐curve to MIC ratio (*f*AUC:MIC) and Monte Carlo simulations. A *f*AUC:MIC ratio target of 72 was used for *E. coli* , *Pseudomonas aeruginosa* , *Pasteurella multocida* , and *Staphylococcus* spp. in accordance with other analyses of fluoroquinolones (Papich et al. 2023; USCAST, The National Antimicrobial Susceptibility Testing Committee for the United States 2018). The “*f*” in the term for AUC value indicates that only the free fraction (protein unbound fraction) of the antimicrobial agent is used in the calculation. The value of 72 essentially represents the average value of the *f*AUC/MIC that produced success in other studies (USCAST, The National Antimicrobial Susceptibility Testing Committee for the United States 2018). For β‐streptococci, we used a lower *f*AUC:MIC ratio target of 33.8, consistent with the value used by others (USCAST, The National Antimicrobial Susceptibility Testing Committee for the United States 2018). To derive this ratio, the following formula was used (Equation 2):

(2) $$\frac{f\mathrm{AUC}}{\mathrm{MIC}}=\frac{\mathrm{fu}\times D}{\mathrm{MIC}\times \mathrm{CL}/F}$$

where *fu* is the unbound fraction of marbofloxacin, enrofloxacin, and ciprofloxacin in feline plasma, D is the dose administered, and CL/F is clearance. For Monte Carlo simulations, the clearance value corrected for bioavailability (F) was used (CL/F); therefore, bioavailability was not included in the equation.

Monte Carlo simulations were performed by generating the *f*AUC based upon the fluoroquinolone CL values (log‐normal distribution) and the associated Std Dev derived from our pharmacokinetic analysis. In addition to CL, we entered the dose, *fu* for enrofloxacin log‐normal distribution (*fu* mean 0.68, Std Dev 0.05) and ciprofloxacin log‐normal distribution (*fu* 0.82, Std Dev 0.013), and the fraction of enrofloxacin *f*AUC that is converted to ciprofloxacin (0.16). Given that a single 5 mg tablet could be used for cats across a range of body weights, simulations were based upon a continuous variable that ranged from 4.5 to 5.5 mg/kg. Monte Carlo simulations (Oracle Crystal Ball version 11.1.3.0.0; www.Oracle.com) calculated the *f*AUC/MIC over a range of MIC values entered.

The marbofloxacin simulations were performed in a similar manner. Regarding marbofloxacin, insufficient data were available to describe a variability in protein binding. As discussed below, our laboratory estimate of marbofloxacin protein binding was approximately zero. However, a sponsor's technical report estimated a protein binding value of 7%. Therefore, given the uncertainty in what constituted a mean population estimate, *fu* was simulated as a continuous function whose value could range between 0.86 and 1.0. For dose, because of the limitations in tablet size available, the mg/kg dose was entered as a continuous variable that could range between 2.5 and 3.06 mg/kg for the 2.78 mg/kg dose and 4.95 and 6.05 mg/kg for the 5.5 mg/kg dose.

The Monte Carlo simulations were generated for 10,000 trials. The MIC values used in the simulation were two‐fold dilutions to include 0.03, 0.06, 0.12, 0.25, 0.5, 1.0, 2.0, 4.0, and 8.0 μg/mL. The PTA threshold for breakpoint development was ≥ 90% for the MIC values, indicating that 90% of the simulated PK values were at or above the threshold considered therapeutically effective for that dose–MIC combination. The 90% value for PTA is the threshold suggested by others (USCAST, The National Antimicrobial Susceptibility Testing Committee for the United States 2018; Ambrose 2006; Turnidge and Paterson 2007).

### 2.4 Microbiology Data

To compare our PK‐PD derived cutoff values (CO_PD_) with microbiologic data (MICs), we obtained data from surveillance programs launched post‐approval in North America (NA). The data from the Centre Européen d'Etudes pour la Santé Animale (CEESA), ComPath program. We also included in our tables data published in other sources (de Jong et al. 2020; Ludwig et al. 2016; Moyaert et al. 2017, 2019; Morrissey et al. 2016; Temmerman et al. 2024). The NA program isolates were collected from a monitoring program that collected data from veterinary diagnostic laboratories throughout the US and Canada from 2011 to 2021. These isolates were limited to skin and soft tissue infections (SSTI) and urinary tract infections (UTI), and from primary care/general care practices only. North American isolates were included regardless of prior antimicrobial therapy. We also included data from the European Committee on Antimicrobial Susceptibility Testing (EUCAST) MIC distribution database (European Committee on Antimicrobial Susceptibility Testing 2020). The complete microbiology data are available in Tables S1–S7.

**FIGURE 1:**
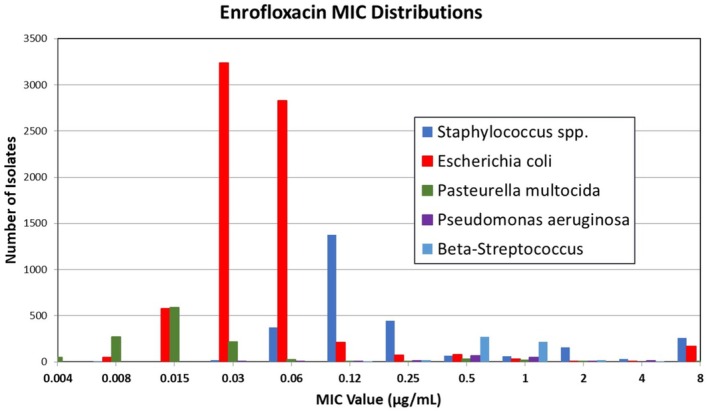
Distribution of enrofloxacin MIC values for selected bacterial isolates.

**FIGURE 2:**
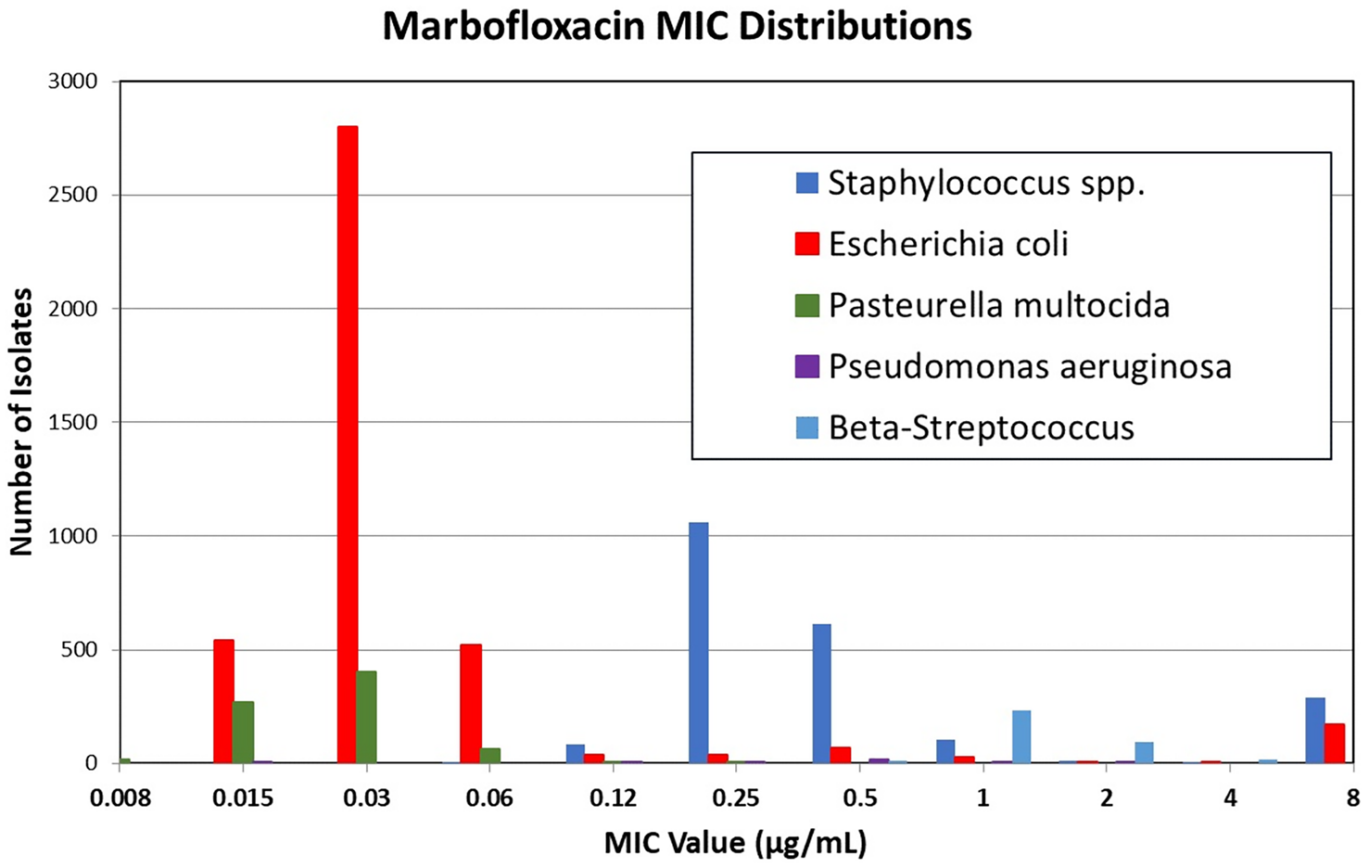
Distribution of marbofloxacin MIC values for selected bacterial isolates.

The Minimal Inhibitory Concentration (MIC) distributions, cumulative percent, and wild‐type cutoff (CO_WT_) values were calculated and included in the Tables with our results. We calculated the CO_WT_ using the “ECOFF Finder”, which is available as a public access tool (https://clsi.org/meetings/susceptibility‐testing‐subcommittees/ecoffinder/). The MIC distributions were also presented as histograms (Figures 1 and 2) that correspond to the data in Tables S1–S7 for each antimicrobial agent and bacterial isolate for visual inspection.

## 3 Results

### 3.1 Pharmacokinetic Data

The pharmacokinetic data from 15 datasets for enrofloxacin and 8 datasets for marbofloxacin, representing 279 and 145 cats, respectively, are presented in Tables S3 and S4, respectively. Because enrofloxacin is partially metabolized to ciprofloxacin, the data collected from other studies (Bregante et al. 2003; Ludwig et al. 2016; Moyaert et al. 2017) determined the overall mean contribution of ciprofloxacin and enrofloxacin to the total fluoroquinolone AUC to be 14% (Std Dev 4) and 85% (Std Dev 4) for ciprofloxacin and enrofloxacin, respectively.

The mean value and associated variability (Std Dev) of CL/F for enrofloxacin was 0.273 L/kg/h (0.078 Std Dev). The CL/F LS mean value for marbofloxacin was 0.101 L/kg/h (0.027 Std Dev). This summary is presented in Table S5.

### 3.2 Protein Binding

We used protein binding results for enrofloxacin from a published source (Bregante et al. 2003). The value used for this analysis was 32.3% (*fu* 0.677 ± 0.051). There was only one protein binding value available for marbofloxacin, which was obtained from the sponsor's technical report. This value was 7.3% (no variation listed). Our independent study used 3 replicates at 3 concentrations. The concentration obtained in the protein‐free filtrate was undetectable. Therefore, the protein binding was essentially 0.0. We averaged these values from each source to obtain a value of protein binding of 4% (*fu* 0.96) for the marbofloxacin Monte Carlo simulations. Variability in protein binding was included in our Monte Carlo simulations. This was included to account for any uncertainty in the population mean value for protein binding.

### 3.3 Monte Carlo Simulations

**FIGURE 3:**
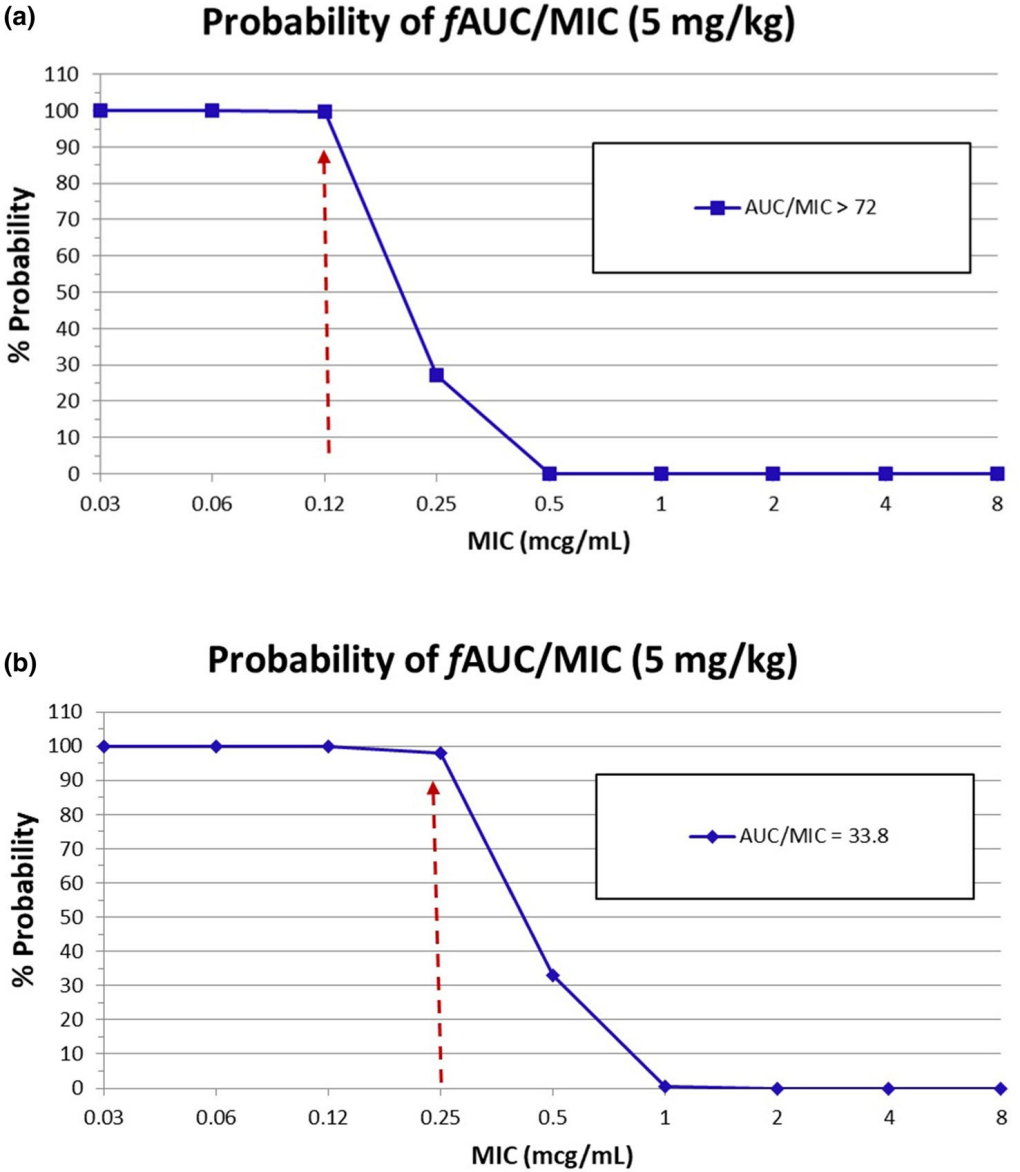
(a) Plot of the Probability of Target Attainment (PTA) for a range of MIC values for Enrofloxacin at a Dose of 5 mg/kg Administered Orally to Cats Once Daily for *Escherichia coli* , *Pseudomonas aeruginosa* , *Staphylococcus* spp., and *Pasteurella multocida* . The arrows indicate the MIC corresponding to a PTA of 90% or greater to reach a target of *f* AUC/MIC > 72 using PK‐PD analysis and Monte Carlo simulations. PTA, probability of target attainment, expressed as a percentage; MIC, minimal inhibitory concentration in μg/mL. (b) Plot of the Probability of Target Attainment (PTA) for a range of MIC values for Enrofloxacin at a Dose of 5 mg/kg Administered Orally to Cats Once Daily for β‐streptococci. The arrows indicate the MIC corresponding to a PTA of 90% or greater to reach a target of *f* AUC/MIC > 33.8 using PK‐PD analysis and Monte Carlo simulations. PTA, probability of target attainment, expressed as a percentage; MIC, minimal inhibitory concentration in μg/mL.

**FIGURE 4:**
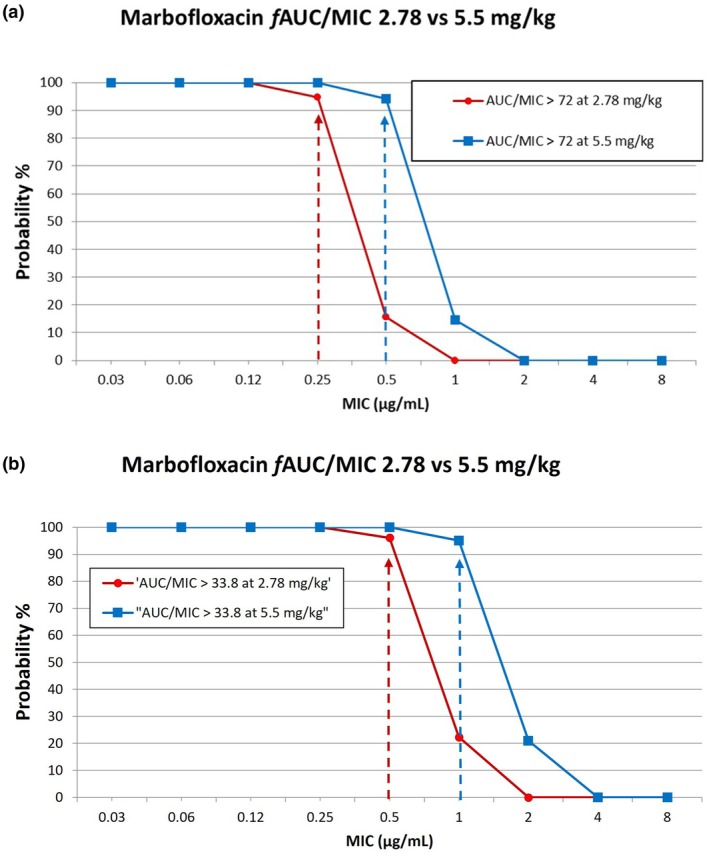
(a) Plot of the Probability of Target Attainment (PTA) for a range of MIC values for Marbofloxacin at 2 doses administered orally to cats once daily for *Escherichia coli, Pseudomonas aeruginosa*, *Staphylococcus* spp., and *Pasteurella multocida* . The arrows indicate the MIC corresponding to a PTA of 90% or greater to reach a target of *f* AUC/MIC > 72 using PK‐PD analysis and Monte Carlo simulations. (b) Plot of the Probability of Target Attainment (PTA) for a range of MIC values for Marbofloxacin at 2 doses administered orally to cats once daily for β‐streptococci. The arrows indicate the MIC corresponding to a PTA of 90% or greater to reach a target of *f* AUC/MIC > 33.8 using PK‐PD analysis and Monte Carlo simulations.

The results from the PK‐PD analysis using Monte Carlo simulation for each dose analyzed are shown in Figures 3 and 4, for enrofloxacin and marbofloxacin, respectively. These figures are derived from the results in Tables S6 and S7 for enrofloxacin and marbofloxacin, respectively. Our threshold for selecting a CO_PD_ is a PTA of approximately 90% or greater. Based on the values in Figure 3a and Table S6 for enrofloxacin, the susceptible CO_PD_ is ≤ 0.12 μg/mL for a dose of 5 mg/kg, oral once daily (Table 1). Because of a lower PK‐PD target for *Streptococcus* spp., the susceptible CO_PD_ is higher by one dilution at ≤ 0.25 μg/mL at the same dose (Figure 3b, Table 1). Based on the results shown in Figure 4a and Table S7 for marbofloxacin, the MIC CO_PD_ is ≤ 0.25 μg/mL for the low dose of 2.8 mg/kg, and ≤ 0.5 μg/mL for a high dose of 5.5 mg/kg (Table 1). This high dose represents a susceptible dose‐dependent (SDD) category that has been used by CLSI for other antimicrobial agents. For *Streptococcus* spp., the CO_PD_ is higher by one dilution at ≤ 0.5 μg/mL at the same dose (Figure 4b, Table 1).

**TABLE 1:** Suggested and current (CLSI) (CLSI 2024) interpretive categories and breakpoint for enrofloxacin and marbofloxacin administered orally to cats.

Antibacterial agent	Interpretive categories and MIC breakpoints, μg/mL			
	S	I	SDD	R
*Escherichia coli* , *Pseudomonas aeruginosa* , *Staphylococcus* spp., and *Pasteurella multocida*				
Enrofloxacin (current)	≤ 0.5	1–2	—	≥ 4
Enrofloxacin (suggested)	≤ 0.12	0.25	—	≥ 0.5
Marbofloxacin (current)	≤ 1	2	—	≥ 4
Marbofloxacin (suggested)	≤ 0.25	—	0.5	≥ 1
*Streptococcus* spp.				
Enrofloxacin (current)	≤ 0.5	1–2	—	≥ 4
Enrofloxacin (suggested)	≤ 0.25	0.5	—	≥ 1
Marbofloxacin (current)	≤ 1	2	—	≥ 4
Marbofloxacin (suggested)	≤ 0.5	—	1	≥ 2

*Note:* The value listed in each cell are the breakpoints (μg/mL) for each interpretive category. The “S” category refers to susceptibility at the lowest label dose of 2.8 and 5 mg/kg for marbofloxacin and enrofloxacin, respectively. The SDD category refers to a higher dose of 5.5 mg/kg for marbofloxacin but is not applied to enrofloxacin because of safety concerns for enrofloxacin in cats.

Abbreviations: I, intermediate; R, resistant; S, susceptible; SDD, susceptible dose‐dependent.

### 3.4 Microbiology Data

We collected data from 1234, 2782, 7273, 155, and 542 feline isolates for *Pasteurella multocida* , *Staphylococcus* spp., *Escherichia coli* , *Pseudomonas aeruginosa* , and β‐streptococci, respectively tested for susceptibility to enrofloxacin. We collected data from 756, 2172, 4234, 34, and 354 feline isolates for *Pasteurella multocida* , *Staphylococcus* spp., *Escherichia coli* , *Pseudomonas aeruginosa* , and β‐streptococci, respectively tested for susceptibility to marbofloxacin. The distributions are presented in Figures 1 and 2, for enrofloxacin and marbofloxacin, respectively. The susceptibility test results, expressed as MICs, are presented in Tables S1 and S2 that correspond to the figures. Included in these tables are the cumulative percent and CO_WT_ (ECOFF values). The CO_WT_ 99.0% for enrofloxacin was 0.06 μg/mL, 0.25 μg/mL, 0.12 μg/mL, 2 μg/mL, and 2 μg/mL for *P. multocida* , *Staphylococcus* spp., *E. coli* , *Pseudomonas aeruginosa* , and β‐streptococci, respectively. The CO_WT_ 99.0% for marbofloxacin was 0.06 μg/mL, 0.5 μg/mL, 0.06 μg/mL, 2 μg/mL, and 2 μg/mL for *P. multocida* , *Staphylococcus* spp., *E. coli* , *Pseudomonas aeruginosa* , and β‐streptococci, respectively.

These distributions provide a comparison between the CO_WT_ value and the PK‐PD‐derived cutoffs (CO_PD_). Although our suggested breakpoints are based solely on the PK‐PD cutoff, the MIC distributions provide a comparison of the proportion of bacteria that falls within each interpretive category.

## 4 Discussion

Our objective was to develop suggested revised breakpoints for these fluoroquinolones that may be considered by laboratories, standard‐setting organizations, and industry sponsors of these antimicrobials for testing common bacteria isolated from cats. We calculated the CO_PD_ values for each fluoroquinolone and dose. Because the CLSI uses the CO_PD_ as the breakpoint under these conditions (CLSI 2021), the values presented in Table 1 can be considered for a clinical breakpoint. These feline breakpoints are needed because when the breakpoints for dogs were revised (Papich et al. 2023), it created a discrepancy between the new canine breakpoints and the currently available feline breakpoints (CLSI 2024). In addition, the new SDD category for enrofloxacin breakpoints in dogs (Papich et al. 2023) allows for the administration of high doses, but those doses cannot be administered to cats because of safety concerns.

The currently used feline breakpoints (CLSI 2024) were established more than 25 years ago. Since then, new pharmacokinetic data are available for these agents in cats. In addition, new microbiologic data have been collected, and we have a better understanding of the PK‐PD relationships appropriate for the development of breakpoints for susceptibility testing of fluoroquinolones. For this study, we used modern methods for analyzing PK‐PD data and applying these for Monte Carlo simulations to predict the best outcome (Martinez et al. 2012; Drusano 2004; USCAST, The National Antimicrobial Susceptibility Testing Committee for the United States 2018; Ambrose 2006; Turnidge and Paterson 2007).

If the proposed revised feline enrofloxacin and marbofloxacin breakpoints are adopted by clinical laboratories, standard‐setting organizations, and industry sponsors, it will help promote safe and effective treatment of cats and support antimicrobial stewardship. Importantly, these suggested feline breakpoints highlight that only those pathogens with low MIC values can be treated at the 5 mg/kg enrofloxacin dose that is restricted to reduce the risk for blindness in cats.

This report provides the data for suggested breakpoints for enrofloxacin and marbofloxacin when used to treat infections in cats caused by *P. multocida* , *Staphylococcus* spp., *E. coli* , *P. aeruginosa* , and *Streptococcus* spp. Those who consider these breakpoints should be aware that most *P. aeruginosa* will not test susceptible using these categories, and it may not be necessary for a laboratory to test all isolates. Likewise, even with a lower PK‐PD target for *Streptococcus* spp., most isolates from cats will not be susceptible, and other more highly active agents such as the beta‐lactam antibiotics are likely to be better choices for treatment. Many *Staphylococcus* spp. will either need a higher dose for marbofloxacin or will not test susceptible to enrofloxacin. Susceptibility testing or selecting a more active antimicrobial agent is encouraged before considering these fluoroquinolones to treat a *Staphylococcus* spp. infection in cats. The MIC distributions of these bacteria are presented in Tables S1–S7. Another consideration for the laboratories is that quality control (QC) enrofloxacin and marbofloxacin data are not available for testing *Streptococcus* spp. and *Pasteurella multocida* . The laboratories will need to ensure that the laboratory test is providing an accurate interpretation before including the results in a report.

We included the variability in protein binding in our Monte Carlo simulations. This accounted for uncertainty in the population mean value for protein binding. However, we also recognize that the inclusion of variability could negatively bias our predictions (Martinez et al. 2025). To confirm the absence of a negative bias in our predictions, we repeated our simulations using only mean *fu* values. The results of our breakpoint predictions did not change, thereby supporting our original predictions.

For enrofloxacin, only one S breakpoint is suggested. Isolates that test in the enrofloxacin Intermediate (I) category should not be treated with higher doses above 5 mg/kg per day in cats because of safety concerns. Marbofloxacin has an approved label that allows for a range of safe doses. Therefore, we suggest an S category for the low dose of 2.78 mg/kg per day, and a new SDD category, similar to other antimicrobials reported by CLSI (Papich et al. 2023), that allows for a higher dose of 5.5 mg/kg per day for attainment of a higher MIC.

In Table 1, these suggested interpretive categories are shown. There are important differences between these suggested breakpoints and those currently published (CLSI 2024). Bacteria from feline infections that may test “susceptible” with the current breakpoint may test “resistant” using these new suggested breakpoints. Compared to the current breakpoints (CLSI 2024), the SDD category for marbofloxacin will require the use of a higher dose to reach therapeutic targets.

Figures 1 and 2 show that although many isolates of *E. coli* and *Staphylococcus* spp. will test in the S category, there are resistant strains with high MIC values in the R category using these suggested breakpoints. This illustrates the value of performing a susceptibility test to determine the potential for a positive therapeutic outcome before either antimicrobial agent is prescribed, or if the cat has not responded to one of these agents.

This analysis was limited to the bacterial species shown in Figures 1 and 2 and in our Tables S1–S7 because of the information available from surveillance programs used to collect our data. However, because other bacteria of the Enterobacterales are expected to respond to the same PK‐PD targets, the breakpoints presented in Table 1 should apply to all bacteria in the Enterobacterales.

There are some limitations to this analysis. There is no guarantee of a clinical cure when a bacterial isolate in the “susceptible” breakpoint category is treated with these agents. There are many factors that contribute to clinical success, including the animal's immune status, organ dysfunction, location of the infection, and concurrent medications, among others. Ultimately, given the range of factors that can influence the clinical response and the large number of patients that would be needed to validate these suggested clinical breakpoints, large prospective studies and post‐marketing evaluation by the sponsors are needed. Our pharmacokinetic data also have limitations. Although these data were derived from 15 data sets and 279 observations for enrofloxacin and 8 datasets and 145 observations for marbofloxacin, these data were obtained from studies performed in healthy animals. Except for the Foster 2023 study (Foster et al. 2023), there were no data available from cats with clinical disease. It is possible that pharmacokinetics may be different in healthy cats compared to cats with infections. Lastly, there is a risk of negative bias being introduced into the clinical breakpoint assessment of enrofloxacin by our inclusion of the variability for the fraction unbound (*fu* in Equation 2) estimate (Toutain et al. 2023). However, additional assessment of the impact of *fu* variability on this bias was found to be influenced by the relative standard deviation (% CV) of CL/F and *fu* (Martinez et al. 2025). Given that the *fu* %CV was 7.5 and that of CL/F was 36.6, we can conclude that negligible bias was introduced by the inclusion of an *fu* standard deviation value into these Monte Carlo simulations.

In summary, this report describes suggested methods that can be considered to revise the breakpoints for testing bacterial isolates from cats for susceptibility to enrofloxacin and marbofloxacin. These breakpoints may be considered by laboratories, standard‐setting organizations, and industry sponsors of these antimicrobials for testing common bacteria isolated from cats. There are some important differences from the current breakpoints. Some bacteria that are considered “susceptible” with the current breakpoint of (CLSI 2024) may test R with our suggested revised breakpoints. For isolates that test in the SDD category, a high dose of marbofloxacin will be needed.

If PK‐PD targets are not achieved after administering antimicrobial agents to animals because the dose is too low or the testing interpretation is incorrect, clinical success is less likely and multi‐drug resistance can emerge, which is a risk to both animal and human health. In the rationale for a revision of the human fluoroquinolone breakpoints (Van et al. 2019) the authors describe these risks and the need to revise outdated breakpoints. Ultimately, this continual review and revision of clinical breakpoints will contribute to antimicrobial stewardship and reduce, whenever possible, the unnecessary or inappropriate use of antimicrobial agents (Ambrose et al. 2020).

## Author Contributions

**Mark G. Papich:** contributed to the collection of pharmacokinetic data, analysis of these data and writing of the manuscript. **Lacie A. Gunnett:** contributed to the collection of the microbiology data, analysis of microbiology data, and writing of the manuscript. **Marilyn N. Martinez:** contributed to the analysis of the data in this study and writing and editing the manuscript.

## Ethics Statement

The authors have nothing to report.

## Conflicts of Interest

Mark G. Papich has had consulting agreements, received gifts, honoraria, and support from Zoetis and Elanco (formerly Bayer). These companies are sponsors of the antimicrobials mentioned in this article. He is an unpaid volunteer for the CLSI. Lacie Gunnett is a Principal Scientist for Global Biologics and an employee of Zoetis, the sponsor of one of the antimicrobials mentioned in this article. Marilyn N. Martinez is a Senior Scientist with the United States Food and Drug Administration. She has no affiliation with any drug sponsors and is an unpaid volunteer for the CLSI. The views expressed in this paper do not reflect an official position of the FDA. The authors are unpaid volunteers for CLSI. However, the analysis and views expressed in this paper do not necessarily reflect those of the CLSI‐VAST subcommittee or management. No AI‐assisted technologies were used in the composition of this manuscript.

## Supporting information


**Table S1:** MIC distributions for Enrofloxacin for isolates collected from feline skin, soft‐tissue, and urinary tract infection samples, and corresponding wild‐type cutoff values.
**Table S2:** MIC distributions for Marbofloxacin for isolates collected from feline skin, soft‐tissue, and urinary tract infection samples, and corresponding wild‐type cutoff values.
**Table S3:** Pharmacokinetic data for enrofloxacin after administration to cats.
**Table S4:** Pharmacokinetic data for marbofloxacin after oral administration to cats.
**Table S5:** Summary of pharmacokinetic data in cats.
**Table S6:** Monte Carlo simulations—enrofloxacin in cats.
**Table S7:** Monte Carlo simulations—marbofloxacin.

## Data Availability

The data that supports the findings of this study is presented in the main body of the manuscript, and available in Supplementary Tables S1–S7 that accompany this article.
