# Supplementary material for: The Need for a Revision of Fluoroquinolone Breakpoints for Interpretation of Antimicrobial Susceptibility Testing of Feline Bacterial Isolates
Source: J Vet Pharmacol Ther. 2025 Sep 29;49(2):131–40. doi: 10.1111/jvp.70028 (PMC12968511; doi:10.1111/jvp.70028)
Supplement: Supplementary file 1 — Table S1: MIC distributions for Enrofloxacin for isolates collected from feline skin, soft‐tissue, and urinary tract infection samples, and corresponding wild‐type cutoff values. Table S2: MIC distributions for Marbofloxacin for isolates collected from feline skin, soft‐tissue, and urinary tract infection samples, and corresponding wild‐type cutoff values. Table S3: Pharmacokinetic data for enrofloxacin after administration to cats. Table S4: Pharmacokinetic data for marbofloxacin after oral administration to cats. Table S5: Summary of pharmacokinetic data in cats. Table S6: Monte Carlo simulations—enrofloxacin in cats. Table S7: Monte Carlo simulations—marbofloxacin. [file JVP-49-131-s001.docx]

**Supplementary Tables**

**THE NEED FOR A REVISION OF FLUOROQUINOLONE BREAKPOINTS FOR INTERPRETATION OF ANTIMICROBIAL SUSCEPTIBILITY TESTING OF FELINE BACTERIAL ISOLATES**

Mark G, Papich DVM, MS, ACVCP^1^, Lacie A. Gunnett, BS^2^, and Marilyn N. Martinez, PhD^3^

1. College of Veterinary Medicine, North Carolina State University, Raleigh, North Carolina, USA
2. Zoetis, 333 Portage Street, Kalamazoo, Michigan 49007
3. Senior Biomedical Research and Biomedical Product Assessment Service (SBRBPAS) Expert, Center for Veterinary Medicine, US Food and Drug Administration, Office of New Animal Drugs, 7500 Standish Place, HFV-100, Rockville, Maryland 20855

**Corresponding Author:**

Mark G Papich

College of Veterinary Medicine

North Carolina State University

1060 William Moore Drive

Raleigh, North Carolina, USA 27607

Email: [mgpapich@ncsu.edu](mailto:mgpapich@ncsu.edu)

**SUPPLEMENTARY TABLES**

**Supplementary Table 1: MIC Distributions for Enrofloxacin for Isolates Collected from Feline Skin, Soft-Tissue, and Urinary Tract Infection Samples, and Corresponding Wild-Type Cutoff Values.**

This table lists the number of isolates with corresponding MIC values (µg/mL), and the Epidemiological Cutoff Values (ECOFF) broken down into percent subsets. Sources of data are referenced in the main article. Data in this table corresponds to Figure 1 in the main article. Cumm. % is the cumulative percent of isolates.

| **MIC µg/mL** | **MIC Distributions** | | | | | | | | | |
| --- | --- | --- | --- | --- | --- | --- | --- | --- | --- | --- |
|  | ***Escherichia coli*** | | ***Pseudomonas aeruginosa*** | | **staphylococci** | | ***β*-streptococci** | | ***Pasteurella multocida*** | |
|  | **N** | **Cumm. %** | **N** | **Cumm. %** | **N** | **Cumm. %** | **N** | **Cumm. %** | **N** | **Cumm. %** |
| ≤0.002 | 0 | 0.00% | 0 | 0.00% | 0 | 0.00% | 0 | 0.00% | 0 | 0.00% |
| 0.004 | 0 | 0.00% | 0 | 0.00% | 0 | 0.00% | 0 | 0.00% | 52 | 4.21% |
| 0.008 | 52 | 0.71% | 0 | 0.00% | 1 | 0.04% | 0 | 0.00% | 272 | 26.26% |
| 0.016 | 575 | 8.62% | 0 | 0.00% | 0 | 0.04% | 0 | 0.00% | 589 | 73.99% |
| 0.03 | 3236 | 53.11% | 1 | 0.65% | 20 | 0.75% | 0 | 0.00% | 219 | 91.73% |
| 0.06 | 2828 | 92.00% | 2 | 1.94% | 374 | 14.20% | 0 | 0.00% | 27 | 93.92% |
| 0.12 | 214 | 94.94% | 3 | 3.87% | 1375 | 63.62% | 1 | 0.18% | 10 | 94.73% |
| 0.25 | 73 | 95.94% | 11 | 10.97% | 443 | 79.55% | 18 | 3.51% | 3 | 94.98% |
| 0.5 | 79 | 97.03% | 70 | 56.13% | 65 | 81.88% | 273 | 53.87% | 29 | 97.33% |
| 1 | 34 | 97.50% | 47 | 86.45% | 60 | 84.04% | 218 | 94.10% | 22 | 99.11% |
| 2 | 6 | 97.58% | 6 | 90.32% | 155 | 89.61% | 22 | 98.15% | 9 | 99.84% |
| 4 | 4 | 97.64% | 14 | 99.35% | 32 | 90.76% | 9 | 99.82% | 0 | 99.84% |
| >4 | 172 | 100.00% | 1 | 100.00% | 257 | 100.00% | 1 | 100.00% | 2 | 100.00% |
| Total | 7273 | 100% | 155 | 100% | 2782 | 100% | 542 | 100% | 1234 | 100% |

| **MIC µg/mL** | **Wild-Type Cutoff Values (CO_WT_), (ECOFFs) listed by** **MIC values (µg/mL),** | | | | |
| --- | --- | --- | --- | --- | --- |
|  | ***Escherichia coli*** | ***Pseudomonas aeruginosa*** | **staphylococci** | ***β*-streptococci** | ***Pasteurella multocida*** |
| **CO_WT_ 95.0%** | 0.06 | 1 | 0.25 | 1 | 0.03 |
| **CO_WT_ 97.5%** | 0.12 | 2 | 0.25 | 1 | 0.03 |
| **CO_WT_ 99.0%** | 0.12 | 2 | 0.25 | 2 | 0.06 |
| **CO_WT_ 99.5%** | 0.12 | 2 | 0.5 | 2 | 0.06 |
| **CO_WT_ 99.9%** | 0.12 | 2 | 0.5 | 2 | 0.06 |

**Table 1, Continued**

**Supplementary Table 2: MIC Distributions for Marbofloxacin for Isolates Collected from Feline Skin, Soft-Tissue, and Urinary Tract Infection Samples, and Corresponding Wild-Type Cutoff Values.**

This table lists the number of isolates with corresponding MIC values (µg/mL), and the Epidemiological Cutoff Values (ECOFF) broken down into percent subsets. Sources of data are referenced in the main article. Data in this table corresponds to Figure 2 in the main article.

| **MIC µg/mL** | **MIC Distributions** | | | | | | | | | |
| --- | --- | --- | --- | --- | --- | --- | --- | --- | --- | --- |
|  | ***Escherichia coli*** | | ***Pseudomonas aeruginosa*** | | **staphylococci** | | ***β*-streptococci** | | ***Pasteurella multocida*** | |
|  | **N** | **Cumm. %** | **N** | **Cumm. %** | **N** | **Cumm. %** | **N** | **Cumm. %** | **N** | **Cumm. %** |
| 0.008 | 26 | 0.61 | 0 | 0 | 1 | 0.05 | 0 | 0 | 17 | 2.25 |
| 0.016 | 539 | 13.34 | 1 | 2.94 | 0 | 0.05 | 0 | 0 | 268 | 37.7 |
| 0.03 | 2802 | 79.52 | 0 | 2.94 | 0 | 0.05 | 0 | 0 | 402 | 90.87 |
| 0.06 | 523 | 91.88 | 0 | 2.94 | 3 | 0.18 | 0 | 0 | 63 | 99.21 |
| 0.12 | 36 | 92.73 | 4 | 14.71 | 83 | 4.01 | 0 | 0 | 2 | 99.47 |
| 0.25 | 35 | 93.55 | 7 | 35.29 | 1062 | 52.9 | 0 | 0 | 4 | 100 |
| 0.5 | 69 | 95.18 | 18 | 88.24 | 611 | 81.03 | 10 | 2.82 | 0 | 100 |
| 1 | 29 | 95.87 | 2 | 94.12 | 104 | 85.82 | 234 | 68.93 | 0 | 100 |
| 2 | 3 | 95.94 | 1 | 97.06 | 14 | 86.46 | 93 | 95.2 | 0 | 100 |
| 4 | 3 | 96.01 | 0 | 97.06 | 3 | 86.6 | 15 | 99.44 | 0 | 100 |
| >4 | 169 | 100 | 1 | 100 | 291 | 100 | 2 | 100 | 0 | 100 |
| Total | 4234 | 100% | 34 | 100% | 2172 | 100% | 354 | 100% | 756 | 100% |

**Table 2, Continued**

| **MIC µg/mL** | **Wild-Type Cutoff Values (CO_WT_), (ECOFFs) listed by MIC values (µg/mL),** | | | | |
| --- | --- | --- | --- | --- | --- |
|  | ***Escherichia coli*** | ***Pseudomonas aeruginosa*** | **staphylococci** | ***β*-streptococci** | ***Pasteurella multocida*** |
| **CO_WT_ 95.0%** | 0.06 | 1 | 0.5 | 2 | 0.06 |
| **CO_WT_ 97.5%** | 0.06 | 1 | 0.5 | 2 | 0.06 |
| **CO_WT_ 99.0%** | 0.06 | 2 | 0.5 | 2 | 0.06 |
| **CO_WT_ 99.5%** | 0.06 | 2 | 1 | 2 | 0.06 |
| **CO_WT_ 99.9%** | 0.06 | 2 | 1 | 4 | 0.12 |

**Supplementary Table 3: Pharmacokinetic Data for Enrofloxacin After Administration to Cats.**

|  | **n=** | **Dose** | **t1/2** | **Vss** | **CL** | **AUC** | **Cmax** | **Tmax** | **Reference** |
| --- | --- | --- | --- | --- | --- | --- | --- | --- | --- |
|  |  | **mg/kg** | **hr** | **L/kg** | **L/kg/hr** | **ug hr/mL** | **mcg/ml** | **hr** |  |
| Mean | 7 | 5 | 6.70 | 2.37 | 0.257 | 20.30 | - | - | Seguin, et al., 2004 ^7^ |
| Std Dev |  |  | 0.80 | 0.43 | 0.054 | 5.20 | - | - |  |
| Mean | 8 | 4.8 | 6.20 | 4.00 | 0.426 | 12.90 | 1.66 | 0.6 | Richez, et al. 1997 ^8^ |
| Std Dev |  |  | 1.42 | 0.85 | 0.204 | 5.94 | 0.3113 | 0.283 |  |
| Mean | 22 | 2.5 | - | - | 0.309 | 8.10 | 1.21 | 1.64 | NADA 140-441 |
| Std Dev |  |  | - | - | 0.107 | 2.81 | 0.33 | 1.69 |  |
| Mean | 15 | 5 | - | - | 0.250 | 19.96 | 2.02 | 1.40 | ANADA 200-517 Test |
| Std Dev |  |  | - | - |  |  |  |  |  |
| Mean | 15 | 5 | - | - | 0.271 | 18.46 | 1.91 | 1.37 | ANADA 200-517 Reference |
| Std Dev |  |  | - | - |  |  |  |  |  |
| Mean | 20 | 5 | - | - | 0.222 | 22.52 | 2.56 | 0.84 | ANADA 200-551 Test |
| Std Dev |  |  | - | - |  |  |  |  |  |
| Mean | 20 | 5 | - | - | 0.232 | 21.58 | 2.35 | 0.88 | ANADA 200-551 Reference |
| Std Dev |  |  | - | - |  |  |  |  |  |
| Mean | 24 | 5 | - | - | 0.222 | 22.51 | 1.72 | 1.71 | ANADA 200-608 Test |
| Std Dev |  |  | - | - |  |  |  |  |  |
| Mean | 24 | 5 | - | - | 0.206 | 24.25 | 2.04 | 0.96 | ANADA 200-608 Reference |
| Std Dev |  |  | - | - |  |  |  |  |  |
| Mean | 24 | 5 | - | - | 0.358 | 13.97 | 1.55 | 1.25 | ANADA 200-680 Test |
| Std Dev |  |  | - | - |  |  |  |  |  |
| Mean | 24 | 5 | - | - | 0.364 | 13.75 | 1.52 | 1.15 | ANADA 200-680 Reference |
| Std Dev |  |  | - | - |  |  |  |  |  |
| Mean | 20 | 4.36 | - | - | 0.240 | 18.20 | 2.16 | 1.10 | ANADA 200-720 Test |
| Std Dev |  |  | - | - |  |  |  | 0.51 |  |
| Mean | 20 | 4.36 | - | - | 0.233 | 18.75 | 2.15 | 0.96 | ANADA 200-720 Reference |
| Std Dev |  |  | - | - |  |  |  | 0.39 |  |
| Mean | 18 | 4.09 | - | - | 0.281 | 14.56 | 1.28 | 2.18 | ANADA 200-737 Test |
| Std Dev |  |  | - | - |  |  |  | 1.54 |  |
| Mean | 18 | 4.09 | - | - | 0.289 | 14.15 | 1.24 | 2.10 | ANADA 200-737 Reference |
| Std Dev |  |  | - | - |  |  |  | 1.42 |  |
| Mean | 18.60 | 4.61 | 6.45 | 3.19 | 0.28 | 17.60 | 1.81 | 1.30 |  |
| Std Dev | 5.41 | 0.68 | 0.35 | 1.15 | 0.06 | 4.51 | 0.42 | 0.47 |  |

**Supplementary Table 3 Legend:** Sources of data sets are indicated by superscript, or the FOI Summary available at [Animal Drugs @ FDA](https://animaldrugsatfda.fda.gov/adafda/views/#/search). T½, elimination half-life; AUC, total area under-the-curve; CL/F, systemic clearance per fraction absorbed; C_MAX_, peak concentration; T_MAX_, time to peak concentration. “n=” lists the number of subjects in each study. Empty cells represent values that were not reported or not calculated.

**Supplementary Table 4: Pharmacokinetic Data for Marbofloxacin After Oral Administration to Cats.**

|  | **n=** | **Dose** | **T ½** | **CL/F** | **AUC** | **C_MAX_** | **T_MAX_** | **Reference** |
| --- | --- | --- | --- | --- | --- | --- | --- | --- |
|  |  | **mg/kg** | **hr** | **L/kg/hr** | **µg hr/mL** | **µg/ml** | **hr** |  |
| Mean | 6 | 2 | 7.15 | 0.09 | 25.49 | 1.97 | 1.94 | Albarellos, et al., 2005 ^15^ |
| Std Dev |  |  | 1.32 | 0.02 | 7.48 | 0.61 | 2.11 |  |
| Mean | 7 | 5.55 | 12.70 | 0.08 | 70.00 | 4.80 | 1.20 | NADA 141-151 |
| Std Dev |  |  | 1.10 |  | 6.00 | 0.70 | 0.60 |  |
| Mean | 24 | 5.55 | - | 0.07 | 81.87 | 7.17 | 0.97 | ANADA 200-586 Test |
| Std Dev |  |  | - |  |  |  | 0.40 |  |
| Mean | 24 | 5.55 | - | 0.07 | 76.15 | 6.74 | 1.09 | ANADA 200-586 Reference |
| Std Dev |  |  | - |  |  |  | 0.36 |  |
| Mean | 24 | 4.55 | - | 0.11 | 40.26 | 2.87 | 2.13 | ANADA 200-733 Test |
| Std Dev |  |  | - |  |  |  | 1.88 |  |
| Mean | 24 | 4.55 | - | 0.11 | 39.79 | 2.90 | 2.43 | ANADA 200-733 Reference |
| Std Dev |  |  | - |  |  |  | 2.08 |  |
| Mean | 18 | 5 | - | 0.14 | 37.00 | 2.67 | 1.14 | ANADA 200-736 Test |
| Std Dev |  |  | - |  |  |  | 1.14 |  |
| Mean | 18 | 5 | - | 0.13 | 39.18 | 2.90 | 0.87 | ANADA 200-736 Reference |
| Std Dev |  |  | - |  |  |  | 0.84 |  |
| **Mean** | **18.13** | **4.72** | **9.93** | **0.10** | **51.22** | **4.00** | **1.47** |  |
| **Std Dev** |  |  |  | **0.03** | **21.29** | **1.99** | **0.60** |  |

**Table 4 Legend:** Sources of data sets are indicated by superscript, which refers to the reference number in text, or the FOI Summary available at [Animal Drugs @ FDA](https://animaldrugsatfda.fda.gov/adafda/views/#/search). T½, elimination half-life; AUC, area under-the-curve; CL/F, systemic clearance per fraction absorbed; C_MAX_, peak concentration; T_MAX_, time to peak concentration. “n=” lists the number of subjects in each study. Empty cells represent values that were not reported or calculated.

**Supplementary Table 5: Summary of Pharmacokinetic Data in Cats.**

This table represents a summary of the pharmacokinetic data used for Monte Carlo Simulations for the clearance value (CL/F). This summary accounts for within-study variability, between study variability, and weighted by the number of animals in each study listed in Supplementary **Tables 3 and 4.**

|  | **Number of data sets** | **Number of observations** | **CL/F**  **Mean**  **(L/kg/hr)** | **CL/F**  **Std.Dev.** | **CV%** |
| --- | --- | --- | --- | --- | --- |
| **Marbofloxacin** | 8 | 145 | 0.101 | 0.027 | 36.74 |
| **Enrofloxacin** | 15 | 279 | 0.273 | 0.078 | 28.41 |

**Supplementary Table 6: Monte Carlo Simulations – Enrofloxacin in Cats.**

This table represents the results from the Monte Carlo Simulations corresponding to Figure 3 in the main article.

The probability of target attainment (PTA) is shown in each cell for a dose of 5 mg/kg simulated for oral administration to cats once daily. The value represents the percentage probability of meeting a pharmacokinetic-pharmacodynamic (PK-PD) target of *f*AUC/MIC ≥ 72 for a 24-hour interval.

.

|  | **MIC (µg/mL)** | | | | | | | | |
| --- | --- | --- | --- | --- | --- | --- | --- | --- | --- |
|  | **0.03** | **0.06** | **0.12** | **0.25** | **0.5** | **1** | **2** | **4** | **8** |
| **Dose: 5 mg/kg oral** | | | | | | | | | |
| **PTA ^a^** | 100 | 99.93 | 97.77 | 28.77 | 0.2 | 0 | 0 | 0 | 0 |
| **PTA ^b^** | 100 | 100 | 100 | 98.01 | 32.91 | 0.46 | 0 | 0 | 0 |

**Table legend:** PTA, probability of target attainment, expressed as a percentage; MIC, minimal inhibitory concentration in µg/mL. ^a.^ PTA for *Escherichia coli, Pseudomonas aeruginosa, Staphylococcus* spp., and *Pasteurella multocida* with a *f*AUC/MIC ratio of 72. PTA ^b^ for β-streptococci with a *f*AUC/MIC ratio of 33.8

**Supplementary Table 7: Monte Carlo Simulations -- Marbofloxacin.**

This table represents the results from the Monte Carlo Simulations corresponding to Figure 4 in the main article.

The probability of target attainment (PTA) is shown in each cell for 2 doses simulated for oral administration to dogs once daily. The value represents the percentage probability of meeting a pharmacokinetic-pharmacodynamic (PK-PD) target of *f* AUC/MIC ≥ 72 for a 24-hour interval.

|  | **MIC (µg/mL)** | | | | | | | | |
| --- | --- | --- | --- | --- | --- | --- | --- | --- | --- |
|  | **0.03** | **0.06** | **0.12** | **0.25** | **0.5** | **1** | **2** | **4** | **8** |
| **2.8 mg/kg oral** | | | | | | | | | |
| **PTA ^a^** | 100 | 100 | 100 | 94.63 | 15.69 | 0 | 0 | 0 | 0 |
| **PTA ^b^** | 100 | 100 | 100 | 100 | 96.1 | 22.31 | 0 | 0 | 0 |
| **5.5 mg/kg oral** | | | | | | | | | |
| **PTA ^a^** | 100 | 100 | 100 | 100 | 94.07 | 14.58 | 0 | 0 | 0 |
| **PTA ^b^** | 100 | 100 | 100 | 100 | 100 | 95.01 | 20.9 | 0 | 0 |

**Table legend:** PTA, probability of target attainment, expressed as a percentage; MIC, minimal inhibitory concentration in µg/mL.^a.^ PTA for *Escherichia coli, Pseudomonas aeruginosa, Staphylococcus* spp., and *Pasteurella multocida* with a *f*AUC/MIC ratio of 72. PTA ^b^ for β-streptococci with a *f*AUC/MIC ratio of 33.8
